# Restenosis of Coronary Arteries in Patients with Coronavirus Infection: Case Series

**DOI:** 10.1155/2023/3000420

**Published:** 2023-02-09

**Authors:** Gulnara Batenova, Lyudmila Pivina, Evgeny Dedov, Altay Dyussupov, Zhanar Zhumanbayeva, Yerbol Smail, Tatyana Belikhina, Laura Pak, Diana Ygiyeva

**Affiliations:** ^1^Semey Medical University, Department of Emergency Medicine, Semey, Kazakhstan; ^2^Pirogov Russian National Research Medical University, Department of Internal Medicine, Moscow, Russia; ^3^Center of Nuclear Medicine and Oncology, Semey, Kazakhstan

## Abstract

**Introduction:**

Coronavirus infection is a risk factor for vascular thrombosis. This is of particular importance for patients undergoing myocardial revascularization since this infection can be a trigger for the formation of restenosis in the area of a previously implanted coronary stent. Understanding the risk factors for stent thrombosis and restenosis is of particular importance in individuals at risk for adverse outcomes. The rarity of such situations makes the present study unique.

**Objective:**

Studying the peculiarities of restenosis and thrombosis of the coronary arteries in patients after coronavirus infection.

**Methods:**

The study was performed in the Department of Cardiovascular Surgery of Emergency Hospital, Semey City, in 2021. We have examined the medical records of 10 consecutive patients with restenosis of coronary arteries after coronavirus infection and 10 matched-by-age patients with similar restenosis of coronary arteries who did not have coronavirus infection as a comparison group. To determine statistically significant differences between independent samples, we calculated the Mann–Whitney *U* test.

**Results:**

The average age of patients was 65.7 years. Only one case was classified as early restenosis (within 8 days of previous revascularization), two cases represented late restenosis, and seven cases were very late restenoses. In 70% of cases, restenosis was localized in the left anterior descending artery, in 30% of cases, it was in the right coronary artery, and in 40% of cases, it was in the left circumflex artery. In comparison with patients who did not have a coronavirus infection, there were statistically significant differences regarding IgG (*P* < 0.001) and fibrinogen (*P*=0.019).

**Conclusion:**

Patients with myocardial revascularization in the past have a higher risk of stent restenosis against the background of coronavirus infection due to excessive neointimal hyperplasia, hypercoagulability, increased inflammatory response, and endothelial dysfunction.

## 1. Introduction

The gold standard for the treatment of coronary artery disease is myocardial revascularization using stents, balloon angioplasty, or coronary artery bypass grafting [1]. Advances in myocardial revascularization, the introduction of drug-eluting stents, and effective antithrombotic therapy have made coronary restenosis rare in recent years [2]. Coronavirus disease 2019 (COVID-19) has changed our point of view about this pathology. This infection promotes thrombosis of arterial vessels and acts as a provoking factor in the development of acute coronary syndrome (myocardial infarction or unstable angina) [3, 4].

At the peak of the COVID-19 pandemic, due to the high burden on the healthcare system and the sharply increased need for resources, the activity of interventional cardiology around the world decreased significantly, reducing the number of cardiac catheterization procedures [5]. However, the need for repeated cardiac surgery for restenosis of coronary vessels increased against the background of coronavirus infection [6]. Understanding the risk factors for stent thrombosis and restenosis is of particular importance in individuals at risk for adverse outcomes, especially in elderly patients with associated diseases.

### 1.1. Aim of Research

Studying the peculiarities of restenosis and thrombosis of the coronary arteries in patients after coronavirus infection.

## 2. Methods

The study was performed in the Department of Cardiovascular Surgery of Emergency Hospital, Semey City, Kazakhstan, from May 2021 to December 2021. We have examined the medical records of ten consecutive patients with restenosis of coronary arteries after coronavirus infection and ten matched-by-age patients with a similar restenosis of coronary arteries who did not have coronavirus infection as a comparison group. In all patients during restenosis, stents of the third generation were installed (Resolute Integrity, Promus Premier, Xience Alpine, BioMatrix, and Orsiro). The clinical data of patients were collected from electronic medical records, including demographics, clinical symptoms and signs, coexisting conditions, imaging findings, laboratory results, and clinical outcomes. Data on previous myocardial revascularization and coronavirus infection were found retrospectively from medical records. For all patients, the diagnosis was made by experienced specialists. All reported events were verified by hospital electronic records from Complex Medical Information System and adjudicated by two cardiologists in consensus. The study was conducted in accordance with the Declaration of Helsinki (as revised in 2013) and approved by the institutional ethical committee of Semey Medical University (reference number 7 from 16.03.2021). Informed consent and permission to publish were provided by the patients.

The study design is prospective single-center case series. To determine statistically significant differences between independent samples, we calculated the Mann–Whitney *U*-test.

## 3. Results

**Case 1 .**

A 65-year-old man was admitted to the hospital on June 23, 2021. He underwent coronary artery stenting twice (in 2016 and 18.01.2021). In May 2021, he suffered from severe COVID-19-associated pneumonia. Coronarography: the right type of coronary circulation. Left anterior descending artery (LAD): 50% stenosis at the orifice, 50–60% stenosis in the proximal part, and occlusion in the area of the previously implanted stent in the distal third with filling of the distal bed through intra-arterial anastomoses. Right coronary artery (RCA): restenosis of 90% of the previously implanted stent in the middle third.

**Case 2 .**

A 69-year-old man was admitted to the hospital on May 12, 2021. He had a myocardial infarction (MI) in 2014, with RCA stenting. COVID-19 in September 2020. Coronarography: the right type of coronary circulation. LAD: with uneven contours and stenosis of the middle third up to 80%. Left circumflex artery (LCx): with uneven contours, stenosis at the orifice up to 60%, and stenosis of the distal third up to 80%. RCA: previously implanted stent, restenosis up to 90% in the distal third.

**Case 3 .**

**Figure 1:**
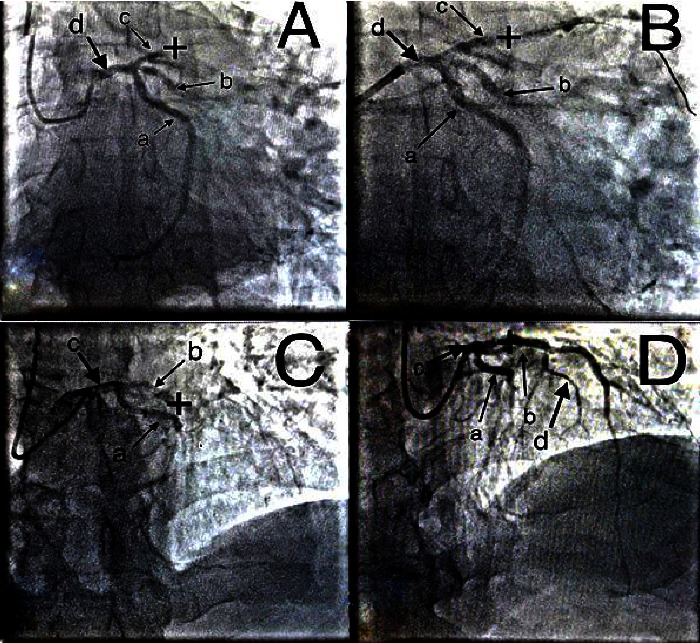
Two cases of acute coronary stent thrombosis.  Case 3: (a) before intervention: a-LCx, b-arteria intermedia, c-LAD, d-left coronary artery, «+»-stent thrombosis; (b) after intervention: «+»-implanted stent. Case 6: (c) before intervention: a-LCx, b-LAD, с-left coronary artery, «+»-stent thrombosis; (d) after intervention: «+»-implanted stent.

A 66-year-old woman was admitted to the intensive care unit on August 23, 2021, with anginal pain and a high level of cardiac markers. In 2013, she suffered from MI and underwent stenting of coronary arteries. In May 2021, she had COVID-19. Coronarography: the right type of coronary circulation. LAD: stent thrombosis of the proximal segment, the distal bed is not visualized. TIMI 0 flow. RCA: midsegment stenosis. TIMI III flow (Figure 1).

**Case 4 .**

A man of 80 years-old was admitted to the intensive care unit on December 3, 2021. In 2017, he suffered from MI, and stenting of the coronary artery was performed. There were manifestations of severe COVID-19-associated pneumonia on a CT scan. The volume of lung damage on the right is 50% and on the left 30%. Coronarography: the right type of coronary circulation. LAD: occlusion of the stented segment in the proximal third, TIMI 0 flow. The patient died after 8 days due to acute left ventricular and respiratory failure.

**Case 5 .**

A man of 66 years-old was hospitalized on 11.09.2021 in the emergency department. In 2012, he underwent MI with coronary artery stenting. In July 2021, he suffered from COVID-19-associated pneumonia. Coronarography: the right type of coronary circulation. LAD: uneven contours, restenosis in a previously implanted stent in the proximal third up to 80%. LCx: 80–90% stenosis in the proximal third followed by occlusion at the border of the proximal and middle thirds, the distal bed is not contrasted, TIMI 0 flow.

**Case 6 .**

A 59-year-old man was hospitalized in the intensive care unit on November 10, 2021. He was hospitalized for the period from 01.11.2021 to 09.11.2021 due to acute anterolateral STEMI and paroxysm of ventricular tachycardia. Stenting (one stent) was carried out on 01.11.2021. In August 2021, he underwent COVID-19. Coronarography: the right type of coronary circulation. LAD: stent thrombosis of the middle segment, the distal bed is not visualized. TIMI 0 flow (Figure 1).

**Case 7 .**

A 71-year-old woman was hospitalized in the intensive care unit with anginal pain on 12.11.2021. In December 2020, she underwent MI with stenting; in the hospital, the patient developed symptoms of respiratory failure, a positive PCR test for COVID-19 was diagnosed, CT scan of the chest showed severe COVID-19-associated pneumonia. After discharge, severe dyspnea persisted at rest, and she received oxygen therapy. Coronarography: the right type of coronary circulation. LAD: 90% restenosis in the previously implanted stent in the proximal third of the orifice, 50% stenosis in the distal third. LCx: stenosis up to 50% at the orifice, distally without significant stenotic lesion.

**Case 8 .**

A 46-year-old man was admitted to the intensive care unit with signs of ACS on 08.11.2021. In 2017, he suffered from anterior MI; stenting was performed. In August 2021, he underwent COVID-19-associated pneumonia. Coronarography: the right type of coronary circulation. LAD: with uneven contours, the stented segment is passable, with restenosis up to 80%. RCA: stenosis 50% in the middle third.

**Case 9 .**

A 70-year-old woman was admitted to the intensive care unit on 07.11.2021. She had hypertension. In 2003, 2005 suffered from MI. In 2007 and 2016, coronary artery stenting was performed. In 2019, episodes of rhythm disorders were registered. In September 2021, she suffered from COVID-19-associated pneumonia. Coronarography: the right type of coronary circulation. LAD: stenosis at the border of the proximal-middle third with narrowing of the arterial lumen up to 70%. LCx: occlusion in the middle third, the distal bed is not contrasted, TIMI 0 flow. RCA: previously implanted stent in the proximal third with 70% restenosis.

**Case 10 .**

A 65-year-old man was admitted to the hospital with anginal pain on 07.12.2021. MI in 2006 and 2008. Coronary artery bypass grafting (CABG) in 2008. In July 2021, he suffered from COVID-19-associated pneumonia. Coronarography: the right type of coronary circulation. Left coronary artery (LCA): contours of the trunk are uneven, stenosis up to 90%. LAD: occluded in the proximal third. RCA: occluded in the proximal third. Bypass: CABG to LCx is occluded. CABG to RCA is occluded.

After predilation, drug-eluting stents were implanted in all patients in the stenosis zone. At the control angiography: the lumen of the arteries was restored and the blood flow through the artery was TIMI III flow.

Clinical characteristics and data of instrumental examination of persons with restenosis who underwent COVID-19 and comparison group are presented in Table 1. The average age of the studied patients was 65.7 years (from 46 to 80 years); in the comparison group, it was 66.2 years. All patients of the studied group were hospitalized for acute coronary artery disease requiring myocardial revascularization; seven of them had STEMI and three had NSTEMI (diagnosis confirmed by ECG), in comparison group situation was the same. Acute coronavirus infection (CVI) was observed only in two patients out of 10 cases (Cases 4 and 7); in one of them, severe COVID-associated pneumonia was diagnosed with a fatal outcome caused by acute heart failure. The second patient had a repeated case of CVI with an interval of 11 months after previous COVID-associated pneumonia against the background of pulmonary fibrosis and chronic respiratory failure of the III degrees, requiring constant oxygen support. The rest of the patients underwent CVI for the previous period from one month (Cases 1 and 9) to 8 months; infection was confirmed by elevated IgG levels (Table 2).

Signs of acute respiratory failure, accompanied by a decrease in oxygen saturation, tachycardia, and tachypnea, were observed in six patients in the studied group. All patients of the studied group received oxygen support. Signs of respiratory distress syndrome were noted in two patients. In the comparison group, decreased oxygen saturation was found in three patients; however this rate was not less than 95%. There were no statistically significant differences in this indicator in the studied groups (*P*=0.218).

Previous myocardial revascularization in the studied group was carried out in terms of 8 days (Case 6) to 14 years. In three patients, the ejection fraction according to echocardiography was intact; in three patients, it was moderately reduced; in the remaining patients, it was decreased. In all cases, restenosis of the coronary arteries was observed; in most cases, its localization was the LAD; in three cases, it was RCA; in four cases, it was LCx. In the comparison group, the predominant localization was LAD (five cases), and in four cases, it was RCA. Decreased injection fraction in this group was found in five cases. There were no statistically significant differences in this rate in the studied groups (*P*=0.65). In all cases, the underlying disease was accompanied by hypertension, and 40% of the patients had type 2 diabetes mellitus.

Analyzing laboratory parameters, all persons of the studied group had confirmation of a past coronavirus infection in accordance with the levels of IgM and IgG (Table 2). In comparison with patients who did not have a coronavirus infection, there were no statistically significant differences regarding IgM (the rate of acute infection); however, such differences were found for IgG (indicator of CVI in the history) (*P*=0.436; *P* < 0.001, respectively).

Lymphopenia and thrombocytopenia characteristic of CVI were noted only in the case of a deceased patient (Case 4); he also had a pronounced leukocytosis, indicating the addition of a bacterial infection. In three cases, moderate leukocytosis was noted against the background of high levels of cardiac markers. A high D-dimer was found only in two cases (one case of acute CVI, the second in a patient who had CVI three months ago). With regard to the lipid profile, almost all patients showed a tendency to increase LDL levels to the upper limit of normal values, while HDL levels were at or below the lower limit of normal. There were not any statistically significant differences in the studied and comparison groups regarding these biochemical rates; however, for the fibrinogen level, such differences were significant (*P*=0.019).

## 4. Discussion

Restenosis is angiographically confirmed a narrowing of the lumen of the coronary artery by more than 50%, localized in the area of a previously implanted stent [7]. Most often, restenosis develops within the first three months after previous revascularization [3]. After six months, the risks of restenosis decrease and the process remains stable; since during this period, stent endothelization and remodeling of the coronary artery wall are completed [4]. The mechanism for early restenosis development is associated with trauma to the vascular wall during device implantation, leading to an inflammatory response accompanied by the migration of neutrophils, monocytes, platelets, and the release of inflammatory mediators [8]. Subsequently, the induction of migration of smooth muscle cells into the intima of the vessel with their accumulation and proliferation of fibroblasts is observed. Increased synthesis of the extracellular matrix causes thickening of the neoadventitia and neointima, narrowing the lumen of the coronary artery in the area of the preimplanted stent [9, 10]. Thus, there is a direct relationship between the development of inflammation, the formation of neointima, and the development of restenosis at the site of the implanted stent [11, 12]. After the damage to the intima of the coronary artery by a stent and increased platelet aggregation, their adhesion and secretion contribute to the induction of migration and proliferation of smooth muscle cells [4].

Coronavirus infection is characterized by an acute systemic inflammatory response that can lead to a cytokine storm. A prothrombotic environment and platelet activation aggravate the situation and increase the risk of thrombosis. An increase in the level of tissue thromboplastin leads to an increase in the level of thrombin and fibrin synthesis with the possible development of disseminated intravascular coagulation [13, 14]. Therefore, coronavirus infection can be a trigger for the formation of restenosis in the area of a previously implanted coronary stent through a complex mechanism, which can cause complications in the long-term period after CVI.

In the article, we presented a description of ten cases of coronary arteries restenosis in patients who had a coronavirus infection in comparison with the matched patients who had restenosis without CVI. In each group, only one case was classified as subacute restenosis, the other cases represented late or very late restenosis.

The available literature sources mainly provide information on cases of acute thrombosis of coronary artery stents after coronavirus infection. Thus, in a study conducted in Spain, a sharp increase in the frequency of stent thrombosis during the peak of the pandemic in March–April 2020 was noted, with a decrease in the frequency of coronary interventions by 38% [15]. These data were later confirmed by the results of other similar studies [6, 16, 17]. A feature of our study is the development of restenosis of previously implanted stents in patients at different times after CVI. Only in two cases, coronavirus infection was present at the time of development of restenosis, of which in one case the patient had severe COVID-associated pneumonia with a fatal outcome, in the second case the patient had a repeated episode of an asymptomatic infection confirmed by a high level of IgM. However, given the low percentage of restenosis of coronary artery stents worldwide, our results suggest a possible association between recent CVI and stent restenosis.

In patients included in our study, the incidence of comorbidities did not differ from the comparison group and other populations of patients [18]. Endothelial dysfunction induced by arterial hypertension, diabetes, or obesity contributes to the proliferation and migration of smooth muscle cells and the formation of neointimal hyperplasia [19].

An increase in the level of fibrinogen in the blood plasma can serve as a predictor of restenosis after endovascular stenting. Fibrinogen, accumulating in the atherosclerotic ally affected intima, penetrates into the arterial wall, where it binds to lipoprotein-*α*, low-density lipoproteins (LDL), which in turn triggers the synthesis of mediators involved in thrombosis. Growth factors, actively produced by platelets and monocytes, have a potentiating effect on hyperplasia and the proliferation of arterial smooth muscle cells, thereby triggering the process of restenosis [20]. In our study, in 60% of patients of the studied group, the fibrinogen level significantly exceeded normal values, and in the other patients, it was the upper limit of normal values, which could be associated not only with coronary heart disease but also with a previous coronavirus infection. In the patients included in our study, the level of lipoproteins practically did not go beyond the limits of normal values, which could be explained by the development of late and very late stent restenoses in them.

## 5. Conclusion

The results of our study suggest that patients with myocardial revascularization in the past have a higher risk of developing stent restenosis against the background of coronavirus infection due to excessive neointimal hyperplasia, hypercoagulability, increased inflammatory response, and endothelial dysfunction. Coronavirus infection can be a trigger for restenosis even in the long-term period after the disease, which is due to the long-term inflammatory process in the vascular wall and vascular remodeling associated with the activation of fibrosis processes. Further research is needed to better understand the mechanisms of restenosis and thrombosis in patients after COVID-19.

## Figures and Tables

**Table 1:** Clinical characteristics and data of instrumental examination of persons with restenosis who underwent CVI.

Case/date of repeat revascularization	Age/gender	Evidence of CVI	Diagnosis	Heart rate	RR	SpO_2_	Date of prior revascularization	CAG: damage	EF	ECG	Comorbidities
*Persons with restenosis who underwent CVI (studied group)*											
1/23.06.2021	65/male	May 2021 pneumonia	STEMI	76	18	97	2016, (5 years)	RCA, LAD	25%	Sinus rhythm, (-+) tooth *T* I, AVL, (−) *T* V5V6	AH, DM
							18.01.2021 (5 months)				
2/12.05.2021	69/male	September 2020	NSTEMI	92	22	95	2014 (7 years)	RCA	35	Sinus rhythm	AH
3/22.08.2021	66/female	May 2021	STEMI	100	26	87	2013 (8 years)	LAD	55%	Sinus rhythm. ST-elevation V1–V5	AH, DM
4/03.12.2021	80/male	Current pneumonia	STEMI	108	32	78	2017 (4 years)	LAD	26%	Sinus rhythm. ST segment elevation in I, aVL, V3–V6	AH, CVA, Paroxysmal AF
5/09.11.2021	66/male	July 2021	STEMI	102	24	92	2012 (9 years)	LCx	34%	Sinus rhythm. QS V1–V3.	AH, DM, CKD
6/10.11.2021	59/male	August 2021	STEMI	96	23	95	2021 (8 days)	LAD	41%	Sinus rhythm. MI of the septal, anterior, lateral wall	AH
7/12.11.2021	71/female	December 2020 pneumonia	STEMI	98	23	95	2020 (11 months)	LAD and LCx	44%	Sinus rhythm. Complete left bundle branch block	AH, CVA, BA, CKD
8/08.11.2021	46/male	August 2021 pneumonia	NSTEMI	67	18	97	2016 (5 years)	LAD	52%	Sinus rhythm	AH
9/07.11.2021	70/female	October 2021	STEMI	70	18	98	2007 (14 years)	LCx	56%	Sinus rhythm AV block 1st degree	AH, CVA, Hypothyroidism
10/07.12.2021	65/male	July 2021	NSTEMI	70	20	97	2008 (13 years)	LCx, RCA	48%	Sinus rhythm	AH, DM

*Persons with restenosis without CVI (comparison group)*											
11/04.11.2021	72/male	No	STEMI	80	18	97	2010 (11 years)	RCA	35%	Sinus rhythm	AH
12/05.11.21	64/female	No	NSTEMI	60	18	98	2019 (2 years)	RCA	52%	Sinus rhythm, transitory left bundle branch block	AH, DM
13/23.07.2021	63/male	No	STEMI	70	20	97	2019 (2 years)	RCA	41%	Sinus rhythm	AH, DM
14/05.11.2021	70/male	No	NSTEMI	80	19	98	September 2021 (2 months)	LAD	61%	Sinus rhythm	AH
15/07.05.2021	67/male	No	STEMI	148	24	95	2020 г (1 year)	LCx	35%	Atrial fibrillation	AH
16/09.10.2021	49/male	No	STEMI	78	21	95	2012 г (9 years)	LAD	56%	Sinus rhythm	AH
17/02.06.2021	65/male	No	STEMI	100	22	96	2020 г (1 year)	LAD	24%	Atrial fibrillation	AH, DM
18/11.05.2021	82/female	No	STEMI	64	20	96	March 2021 (2 months)	LAD	56%	Sinus rhythm	AH
19/15.06.2021	72/male	No	STEMI	70	21	95	May 2021 (1 month)	RCA	32%	Atrial fibrillation	AH
20/09.06.2021	57/male	No	NSTEMI	92	21	97	2017 (4 years)	LAD	50%	Sinus rhythm	AH

BA-bronchial asthma; CVI-coronavirus infection; RR-respiratory rate; CAG-coronary angiography; EF-ejection fraction; ECG–electrocardiography; STEMI-ST-elevation myocardial infarction; RCA-right coronary artery; LAD-left anterior descending artery; EAH-electrical axis of the heart; AH-arterial hypertension; DM-diabetes mellitus; NSTEMI- non-ST-elevation myocardial infarction; LCx-left circumflex artery; CKD-chronic kidney disease.

**Table 2:** Laboratory indicators characterizing the presence of recurrent stent thrombosis in persons who underwent CVI.

Case nos.	IgM <2	IgG <10	LEU (3.4–10.0	NEUT (39.0–75.0)	LYM (17.0–48.0)	PLT (150–375)	D-dimer(0.0–550.0)	Troponin (0.008–0.029)	CK (32.0–294.0)	CK-МВ (0.0–25.0)	Fibrinogen (2.0–3.93)	Tg (0.34–1.7)	LDL-beta(0.10–3.0)	HDL alpha (1.0–2.60)
*Persons with restenosis who underwent CVI (studied group)*														
1	0.50	300	7.3	75.3	22.9	155	418	1.432	189	41.5	4.18	2.31	2.88	0.93
2	0.12	87.5	6.8	74.3	22.5	282	489	0.34	204	26	3.9	2.51	2.76	1.2
3	0.75	94.18	14.4	80.6	18.1	546	1411.0	4.854	71	46.1	8.07	1.32	2.56	0.95
4	3.1	28.2	17.2	80.2	8.4	134	4320	38.02	2121.0	231.7	6.2	1.45	2.89	1.01
5	0.21	37.23	13.20	64.8	33.0	255	799.9	40.0	2413.0	380.8	2.85	3.93	2.79	1.06
6	0.65	52.15	7.8	66.7	22.1	349	361.8	39.063	1196.0	120.9	4.32	1.54	2.99	0.9
7	2.77	128.3	12.6	78.0	20.5	243	434.7	6.030	100	21.4	5.44	1.43	2.18	1.08
8	0.28	76.66	9.1	57.9	39.3	154	492.5	0.052	72	21	3.64	1.98	2.18	1.12
9	0.48	86.43	9.2	57.9	39.5	203	216	3.094	195	30.7	3.53	1.72	2.82	1.06
10	0.18	96.2	7.8	74.3	20.1	183.4	192.0	0.031	234.0	24.8	6.7	1.66	3.54	0.91

*Persons with restenosis without CVI (comparison group)*														
11	0.11	4.15	11.7	76.2	21.9	209	980.0	0.694	56	9.9	4.4	1.93	2.23	0.83
12	0.16	2.59	11.4	78.5	20.2	252	175	0.004	57	20	3.53	1.4	1.9	1.01
13	0.6	8.33	11.5	88.2	8.4	191	432	40	484	46.9	4.18	1.8	3.68	1.1
14	0.8	4.8	6.85	39.7	44.6	279	436	0.002	98	18	3.7	1.04	0.79	0.98
15	0.33	0.82	18.6	65.9	31.9	223	2153	40	16128	1612	2.3	2.34	2.4	0.97
16	1.1	6.4	14.8	79	12	216	480	5.99	668	70	3.9	1.36	2.22	1.01
17	0.86	4.8	13.2	88.1	5.5	185	280	3.23	1070	80	2.3	1.1	3.1	0.5
18	0.98	7.86	8.1	76.8	16.7	214	800	14.9	863	69	3.4	1.3	2.27	1.31
19	0.88	6.9	6.7	58.1	30.1	239	970	1.2	1727	215	2.4	1.2	3.68	0.39
20	1.6	6.98	8.91	57.5	34.3	179	256	0.005	80	37	2.7	5.53	3.7	0.69
*P*	0.436	**<0.001**	0.796	0.739	0.529	0.971	1.0	0.604	0.739	1.0	**0.019**	0.190	0.853	0.684

LEU-leucocyte;NEUT-neutrophils;LYM-lymphocyte;PLT-thrombocyte;CK-creatine kinase; CK-МВ-creatine kinase MB; Tg-triglycerides; LDL-beta-low-density lipoprotein-beta; HDL alpha-high-density lipoprotein alpha. The bold value means result has statistical significance.

## Data Availability

The data used to support the study are included in the paper.

## Additional Points

Small sample size and single-center study.
